# Two-Center Evaluation of Disinfectant Efficacy against Ebola Virus in Clinical and Laboratory Matrices

**DOI:** 10.3201/eid2401.170504

**Published:** 2018-01

**Authors:** Sophie J. Smither, Lin Eastaugh, Claire Marie Filone, Denise Freeburger, Artemas Herzog, M. Stephen Lever, David M. Miller, Dana Mitzel, James W. Noah, Mary S. Reddick-Elick, Amy Reese, Michael Schuit, Carly B. Wlazlowski, Michael Hevey, Victoria Wahl-Jensen

**Affiliations:** Defence Science and Technology Laboratory, Porton Down, UK (S.J. Smither, L. Eastaugh, M.S. Lever);; National Biodefense Analysis and Countermeasures Center, Frederick, Maryland, USA (C.M. Filone, D. Freeburger, D.M. Miller, D. Mitzel, J.W. Noah, M.S. Reddick-Elick, A. Reese, M. Schuit, C.B. Wlazlowski, M. Hevey, V. Wahl-Jensen);; Censeo Consulting Group, Seattle, Washington, USA (A. Herzog)

**Keywords:** Ebola virus, filovirus, viruses, disinfection, disinfectant efficacy, bleach, peracetic acid, two-center evaluation, clinical matrix, laboratory matrix

## Abstract

Ebola virus (EBOV) in body fluids poses risk for virus transmission. However, there are limited experimental data for such matrices on the disinfectant efficacy against EBOV. We evaluated the effectiveness of disinfectants against EBOV in blood on surfaces. Only 5% peracetic acid consistently reduced EBOV titers in dried blood to the assay limit of quantification.

Effective disinfection of Ebola virus (EBOV) in body fluids is critical for emergency response to outbreaks. However, for such fluids, data are scarce for disinfectant efficacy. This information is essential for informed disinfection processes, environmental decontamination, waste disposal practices, and safety practices for healthcare workers and public health responders (*1*). We investigated the efficacy of disinfectants against EBOV spiked into cell culture medium and whole blood. 

## The Study

Six disinfectants were tested: Purrell Advanced (GOJO Industries, Akron, OH, USA) 30 μL; Steriplex SD (sBiomed LLC, Orem, UT, USA) 100 μL; Micro-Chem Plus (National Chemical Laboratories, Inc., Winona, MN, USA) 30 μL; Micro-Chem Plus 100 μL; bleach (Clorox, Oakland, CA, USA) 30 μLand 100 μL; acidified bleach 100 μL; and peracetic acid (Sigma-Aldrich, St. Louis, MO, US) 100 μL. The 2 most effective disinfectants were evaluated at 2 laboratories: the National Biodefense Analysis and Countermeasures Center (NBACC; Frederick, MD, USA); and the Defence Science and Technology Laboratory (DSTL; Porton Down, UK). Because of local regulations and operating procedures, some methods were modified between the laboratories (Table 1).

**Table 1:** Disinfection study parameters for evaluation of 6 disinfectants for reducing Ebola virus titers in dry blood or cell culture medium*

Parameter	NBACC	DSTL
Virus (dilution in blood)	Ebola virus/Makona-C05 (1:10)	Ebola virus/Makona-C07 (1:10)
Cells	Vero C1008, Vero 76, clone E6, Vero E6 (ATCC CRL-1586)	Vero C1008, ECACC #85020206
Blood source	Human whole blood in EDTA (Bioreclamation IVT, Westbury, NY, USA)	Fresh whole blood in EDTA from male Porton rats
Blood droplet size (µL) and state	10, wet or dried	20, dried
Surface coupons	304 stainless steel (Diamond Perforated Metals, Visalia, CA, USA); 6061 Aluminum (Speedy Metals, New Berlin, WI, USA) used for experiments with dry medium (peracetic acid and acidified bleach) and wet blood (peracetic acid) only	3014 stainless steel
Disinfectant		
Sodium hypochlorite		
Supplier	Clorox (Oakland, CA, USA)	Sychem (Leigh On Sea, UK)
Concentration tested, vol/vol	0.5%, 10% hypochlorite	1% hypochlorite
Volume tested, µL	30 or 100	100
Contact time, min	15	15
Peracetic acid		
Supplier	Sigma-Aldrich (St. Louis, MO, USA)	Sigma-Aldrich
Concentration tested, vol/vol	5%	0.2%
Volume tested, µL	100	100
Contact time, min	5	10
Micro-Chem Plus		
Supplier	National Chemical Laboratories, Inc. (Winona, MN, USA)	ND
Concentration tested, vol/vol	1.5%	
Volume tested, µL	30 or 100	
Contact time, min	10	
Purell Advanced		
Supplier	GOJO Industries (Akron, OH, USA)	ND
Concentration tested, vol/vol	70% ethanol	
Volume tested, µL	30	
Contact time, min	2	
Steriplex SD		
Supplier	sBioMed LLC (Orem, UT, USA)	ND
Concentration tested	0.015% silver, 10.000% ethanol, 0.020% H_2_O_2_, 0.150% peroxyacetic acid, 0.150% acetic acid, 0.075% inert food grade ingredients proprietary, 89.590% water	
Volume tested, µL	100	
Contact time, min	5	
Acidified bleach		
Supplier	Clorox	ND
Concentration tested, vol/vol	10% bleach (0.5% sodium hypochlorite) + 1% acetic acid	
Volume tested, µL	100	
Contact time, min	15	
Method		
Neutralization	5 mL cell culture medium (10% FBS) or direct recovery using washing/filtration	2 mL cell culture medium (2% FBS)
Recovery method	30 μL bleach: none (5 mL medium only); 100 µL bleach; Steriplex SD: acidified bleach or peracetic acid; after neutralization in 5 mL medium, 2 mL was ultrafiltered in an Amicon (Bedford, MA, USA) 100-kDa NMWL unit, washed 3 times with 2 mL PBS after centrifugation at 5,000 × *g* for 10 min, and resuspended in 2 mL medium; Purell Advanced: diluted to 130 μL with PBS and recovered using a PD MultiTrap G-25 96-well microplate column array. Dilute supernatant to 5 mL with medium; Micro-Chem Plus: samples directly added to Amicon Ultra-0.5 (30- or 100-kDa NMWL) ultrafiltration unit, washed 3 times with 0.5 mL PBS by centrifugation at 5,000 × *g* for 10 min, and resuspended in 5 mL medium	Washed by centrifugation 8,000 rpm for 5 min and resuspended in 1 mL medium

*Titers were assessed by using the 50% tissue culture infectious dose assay. ATCC, American Type Culture Collection; DSTL, Defence Science and Technology Laboratory; ECACC, European Collection of Authenticated Cell Cultures; FBS, fetal bovine serum; NBACC, National Biodefense Analysis and Countermeasures Center; ND, no data; NMWL, nominal molecular weight limit; PBS, phosphate-buffered saline.

At NBACC, material was prepared as follows. Passage 1 of Ebola virus H.sapiens-tc/GIN/2014/Makona-C05 virus (Rocky Mountain Laboratory, National Institutes of Health, Hamilton, MT, USA) was used to generate passage 2 virus stock in Vero E6 cells (Table 1). All work with viable EBOV was performed in Biosafety Level 4 laboratories.

At DSTL, material was prepared as follows. Passage 4 of Ebola virus H.sapiens-wt/GIN/2014/Makona-C07 virus (Public Health England, London, UK) was passed twice in Vero E6 cells, creating passage 6 material. All work with viable EBOV was performed in Biosafety Level 4 laboratories.

Stainless steel and aluminum coupons (≈22 mm^2^) were sterilized before use. Test matrices included cell culture medium, human whole blood (NBACC), or rat whole blood (DSTL).

To test disinfectants, we spiked Ebola/Mak 1:10 into a test matrix and then deposited it onto coupons. We disinfected coupons immediately (wet) or kept them at ambient conditions until dry by visual examination (*2*) and then disinfected (dry). Disinfectants were applied for various contact times (Table 1). We performed no surface agitation or mixing before sample recovery, per the method of the American Society for Testing Materials International (West Conshohocken, PA, USA) (*2*). We performed neutralization by submersion into cell culture medium and vortexing according to recommendations of ASTM International (*2**,**3*) (Table 1). Viable virus was measured in samples as described (*4**,**5*). We performed statistical analysis of sample results (Technical Appendix).

All disinfectants tested reduced virus titer to the assay lower limit of quantification (LLOQ) of the assay when evaluated with EBOV/Mak deposited on surfaces in cell culture medium (Table 2). However, only 5% peracetic acid consistently reduced the titer of EBOV/Mak in dried human blood to the assay LLOQ. These data collectively indicate that surface-dried whole blood provides a more protective matrix for EBOV/Mak than does surface-dried cell culture medium.

**Table 2:** NBACC studies of evaluation of 6 disinfectants for reducing Ebola virus titers in dry blood or cell culture medium*

Disinfectant and volume, µL	Dried blood				Dried cell culture medium			
Mean log_10_ TCID_50_ (SD) disinfected	Mean log_10_ TCID_50_ (SD) control	log difference	*t*-test p value	Mean log_10_ TCID_50_ (SD) disinfected	Mean log_10_ TCID_50_ (SD) control	log difference	*t*-test p value
Purell Advanced, 30	3.1 (0.2)	2.6 (0.3)	<0	9.5 × 10^–1^	0.7 (0)	2.4 (0.3)	1.7	5.6 × 10^–3^
Steriplex SD, 100	2.4 (0.2)	3.0 (0.2)	0.6	8.8 × 10^–3^	0.7 (0)	3.3 (0.2)	2.6	5.9 × 10^–4^
Micro-Chem Plus, 30	2.8 (0.5)	3.4 (0.5)	0.6	4.9 × 10^–2^	0.7 (0)	1.5 (0.2)	0.8	3.6 × 10^–5^
Micro-Chem Plus, 100	1.5 (0.1)	2.9 (0.2)	1.4	1.4 × 10^–4^	ND	ND	ND	ND
Bleach, 30	2.2 (0.5)	3.1 (0.1)	0.9	1.1 × 10^–3^	0.7 (0)	2.7 (0.2)	2.0	1.3 × 10^–3^
Acidified bleach, 100	1.7 (0.3)	2.7 (0.3)	1.1	5.9 × 10^–3^	0.7 (0)	2.6 (0.2)	1.9	2.0 × 10^–3^
Peracetic acid, 100	0.7 (0)	2.7 (0.3)	2.0	4.3 × 10^–3^	0.7 (0)	1.9 (0.4)	1.2	7.8 × 10^–2^

*NBACC, National Biodefense Analysis and Countermeasures Center; ND, no data; TCID_50_, 50% tissue culture infectious dose.

**Figure 1:**
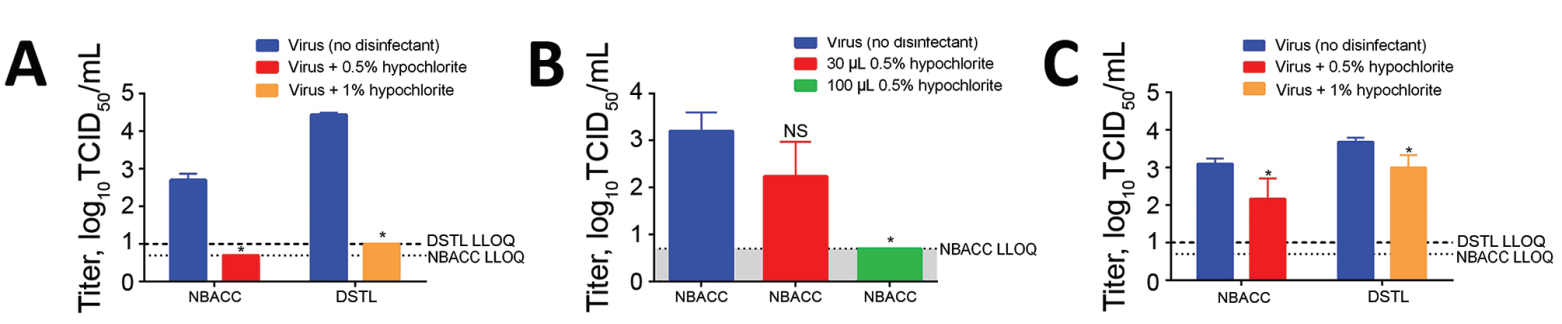
Effect of common bleach disinfection of Ebola virus in A) dried cell culture medium, B) wet blood, and C) dried blood. Coupons were spotted with Ebola virus/Makona (EBOV/Mak). Bleach solutions (0.5% or 1.0% hypochlorite) were effective in reducing the titer of EBOV/Mak to the assay LLOQ in dried cell culture medium or wet blood. Incomplete disinfection was observed when virus was suspended in blood and dried for 1 h before disinfection. Data were confirmed for dried cell culture medium and dried blood studies at an independent laboratory (DSTL). Error bars indicate SD. *Significant difference (p<0.05) between control and disinfected samples. DSTL, Defence Science and Technology Laboratory; LLOQ, lower limit of quantification; NBACC, National Biodefense Analysis and Countermeasures Center.

Because EBOV/Mak in dried blood resists complete bleach disinfection (0.5% or 1.0% sodium hypochlorite) but this method is commonly used in outbreak and healthcare settings (*6*), we sought independent confirmation. We shared protocols and surface coupons with DSTL, who verified that EBOV/Mak dried in cell culture medium was highly susceptible to disinfection with sodium hypochlorite (Figure 1, panel A). However, when dried blood samples were treated with 0.5% or 1.0% sodium hypochlorite, viral titer reductions of only 88.3% and 79.0%, respectively, were observed (Figure 1, panel C). Furthermore, viable virus was recovered from all samples, confirming that dried blood represents a challenging matrix for disinfection of EBOV/Mak.

We also measured effectiveness of disinfection with 10% bleach against EBOV in wet blood on coupons. When we suspended EBOV/Mak in wet blood, 10% bleach was either 89.2% (30 μL) or 99.7% (100 μL) effective in reducing viral titers, depending on the volume (and consequently the final concentrations) of bleach used. Application of 100 μL of bleach to wet blood samples resulted in a significant reduction in viral titer to the LLOQ of the assay (Figure 1, panel B), suggesting that wet blood is less challenging to disinfection than dried blood.

**Figure 2:**
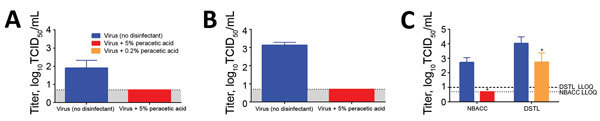
Effect of 5% peracetic acid disinfection of Ebola virus in 3 different matrices. Coupons were spotted with Ebola virus/Makona (EBOV/Mak) in cell culture medium (A) or blood (B, C). Peracetic acid was effective in reducing the titer of EBOV/Mak to the assay LLOQ in dried cell culture medium or wet blood. Although complete disinfection was observed when virus was suspended in blood and dried for 1 h before disinfection with 5% peracetic acid (NBACC), incomplete disinfection was observed with 0.2% peracetic acid (DSTL). Error bars indicate SD. *Significant difference (p<0.05) between control and disinfected samples. DSTL, Defence Science and Technology Laboratory; LLOQ, lower limit of quantification; NBACC, National Biodefense Analysis and Countermeasures Center.

Of the 6 disinfectants we evaluated, only 5% peracetic acid was efficacious in disinfecting dried blood samples containing EBOV/Mak (Table 2). Concentrations of 5% peracetic acid also reduced viral titers to the LLOQ of the microtitration assay when virus was in either dried cell culture medium (Figure 2, panel A) or wet blood (Figure 2, panel B). Although studies at NBACC showed complete inactivation of EBOV/Mak in dried blood by 5% peracetic acid, complementary studies at DSTL showed that use of a lower concentration (0.2%) of peracetic acid resulted in a 94.9% reduction in viral titers in samples with viable virus still present (Figure 2, panel C). Taken together, these results suggest that there might be a concentration-dependent reduction in viral titers in dried blood when peracetic acid is used for disinfection.

## Conclusions

The purpose of this study was to test products for disinfection of EBOV in a relevant clinical matrix. Previous studies showed that filoviruses remain viable in blood for extended periods (*5**,**7**,**8*). Therefore, it was imperative to identify efficacious disinfectants for this matrix. Our results indicate that although bleach, Purell Advanced, and Micro-Chem Plus effectively inactivated EBOV in cell culture medium and wet blood, they were less effective in dried blood.

Only 5% peracetic acid consistently reduced EBOV titers in dried blood to the assay LLOQ. Peracetic acid is a strong oxidant and broad-spectrum disinfectant commonly used in disinfection of a variety of pathogens in waste water because of its relative ease of implementation, broad-spectrum activity in the presence of heterogeneous organic matter, small pH dependence, short contact time, and lack of harmful decomposition products (*9*). Although peracetic acid has been reported to be an effective disinfectant against EBOV (*10*), use of peracetic acid for EBOV disinfection in clinical fluids has not been specifically documented.

Organic matter in clinical fluids can reduce the virucidal activity of disinfectants by a chemical reaction between the disinfectant and the organic matter, which leaves less active disinfectant available for virus inactivation. In particular, chlorine disinfectants are prone to inactivation by reactions with organic matter (*11**,**12*). Alternatively, organic matter can prevent inactivation of viruses by acting as a physical barrier (*13**,**14*). Our results suggest that under the conditions tested, dried blood inhibits effective disinfection of EBOV and might provide a protective layer of matrix not completely dissolved in disinfectant, thereby shielding virus from inactivation. 

This study used the American Society for Testing Materials International standard (*2*) for testing of disinfectants on carriers and represents a worst-case scenario. However, it is possible that precleaning, agitation, or mixing would aid in the disinfection process. Additional testing is required to confirm this possibility.

In summary, our results show the difficulty in disinfecting surfaces contaminated with EBOV in dried blood. Although all disinfectants tested were effective against dried cell culture medium containing EBOV, only 5% peracetic acid reduced dried blood virus titers to undetectable levels. These findings can be used to support public health efforts, risk assessment development, remediation decisions, and response and preparedness procedures for future outbreaks of infection with EBOV.

Technical Appendix Additional information on 2-center evaluation of disinfectant efficacy against Ebola virus in clinical and laboratory matrices.
