# Supplementary material for: Two-Center Evaluation of Disinfectant Efficacy against Ebola Virus in Clinical and Laboratory Matrices
Source: Emerg Infect Dis. 2018 Jan;24(1):135–9. doi: 10.3201/eid2401.170504 (PMC5749448; doi:10.3201/eid2401.170504)
Supplement: Technical Appendix — Additional information on 2-center evaluation of disinfectant efficacy against Ebola virus in clinical and laboratory matrices. [file 17-0504-Techapp-s1.pdf]

Article DOI: https://doi.org/10.3201/eid2401.170504

Two-Center Evaluation of Disinfectant Efficacy against Ebola Virus in Clinical and Laboratory Matrices

Technical Appendix

Statistical Analysis

To identify significant differences between controls and disinfected samples, a 1-sided *t*-test was performed for which $p<0.05$ indicated that disinfected samples contained significantly lower titers than control samples. In instances in which no virus was detected after disinfection, values were considered as left-censored data points that were less than or equal to the assay lower limit-of-quantification (LLOQ); the LLOQ was either 0.7 or 1.2 log 50% tissue culture infectious dose$_{50}$/milliliter, depending on the disinfectant/method. When all replicates for a particular treatment resulted in data points at the LLOQ, an upper-tailed *t*-test was used to test the hypothesis that the control mean was greater than the LLOQ:

$$H_0: \mu_{\text{control}} - \text{LLOQ} \le 0$$
$$H_1: \mu_{\text{control}} - \text{LLOQ} > 0$$

That is, if all data are observed to be at or below the LLOQ, then the LLOQ is significantly different than the mean of observed control data. All statistical analyses were performed by using MATLAB version 2015b (https://www.mathworks.com/company/newsroom/mathworks-announces-release-2015b-of-the-matlab-and-simulink-product-families.html).
